# The Impact of Acoustic Synthetic Jet Actuator Parameters on the Generated Noise

**DOI:** 10.3390/mi16070803

**Published:** 2025-07-10

**Authors:** Emil Smyk, Michał Stopel

**Affiliations:** Faculty of Mechanical Engineering, Bydgoszcz University of Science and Technology, Al. Prof. S. Kaliskiego 7, 85-796 Bydgoszcz, Poland; michal.stopel@pbs.edu.pl

**Keywords:** sound pressure level, jets, turbulence, zero-net-mass flow

## Abstract

Synthetic jet actuators are becoming increasingly popular for enhancing electronic heat transfer. However, their use is currently limited due to the high noise they generate. This article examines how actuator parameters (orifice diameter, orifice length and cavity height) affect synthetic jet velocity and noise generation. Hot-wire anemometry was used to measure velocity, and noise was measured with a sound meter. The actuator was supplied with constant power at different frequencies ranging from 50 to 500 Hz. Observation of the velocity showed that it decreased with an increasing orifice diameter and increased with a decreasing orifice length. No relationship was observed between cavity height and synthetic jet velocity. This article indicates that increasing the orifice diameter or reducing the orifice length causes an increase in the noise generated by SJAs, provided we remain in the vicinity of the characteristic frequency. It was demonstrated that higher actuator chambers produce higher noise levels, although this was not a consistent trend across the entire tested frequency range.

## 1. Introduction

The impact of noise on human well-being has been investigated repeatedly [1,2]. Therefore, the level of noise generated may determine whether certain devices can be used in certain spaces. It should therefore be considered an important parameter, particularly in the case of equipment operating in the presence of humans [3,4]. One such device is the synthetic jet actuator (SJA), which can be used in offices to cool electronics, such as LED lamps [5].

A synthetic jet (SJ) is produced by periodically sucking and blowing the surrounding fluid through an orifice. Vortex rings are induced on the edges of the blown fluid. The vortex ring and turbulent nature of SJs make them useful in a wide range of scientific and technological applications. Gil et al. [5] used an SJ to cool a 150 W LED lamp. An SJA was a part of the heat exchanger and was used to force airflow in a heat sink. The authors are planning to use the presented SJ heat exchanger to cool a 500 W LED lamp. Ding et al. [6] used an SJ for active flow control. The use of an SJ in a flow around a rectangular column allowed for a reduction in the aerodynamic drag coefficient and an increase in the lift coefficient. Murillo-Rincón and Duque-Daza [7] used an SJ to modulate the noise generated by turbulent flow. Applications of SJs have been described and discussed in more detail by Ja’fari et al. [8].

SJAs are devices that generate significant noise due to their method of operation and the turbulent nature of the generated flow. One of the first studies to address the investigation of and reduction in noise generated by actuators was an article published in 2007 by Arik [9]. He closed an SJA in a box with a muffler of different sizes. The use of the muffler decreased The sound pressure level (SPL) by up to 40 dB. Lasance et al. [10] investigated the impact of the orifice diameter and length on the noise level for twin SJs in 2008. The SPL(A) level decreased with the increase in orifice length and the decrease in orifice diameter. Additionally, they showed that an SJA can generate lower noise than a fan and better heat transfer enhancement.

Gil et al. [11] investigated multiple-orifice SJAs with different numbers and diameters of orifices. They stated that the SPL(A) is dependent on the orifice diameter-to-diaphragm diameter ratio (considering the number of orifices), and the highest noise was obtained for a ratio equal to 0.2. Arafa et al. [12] investigated an SJA with different orifice configurations but with the same area. They showed that the SPL depends on the SJ velocity. Generally, the higher the velocity, the higher the SPL. This observation was presented earlier by Gil and Wilk [13]. However, they showed that the noise change with increasing speed is significantly influenced by the used loudspeaker (in the case of acoustic SJAs) and the input power of the actuator. On the other hand, the shape of the SJA orifice also has a significant impact on the generated noise. Smyk et al. [14] reported that the orifice generating the lowest SPL(A) is the square orifice, followed by the circular one, whereas the slot orifice is the loudest, at the same actuator input power. However, it was not indicated that this dependence was due to velocity. Additionally, Smyk and Markowicz [15] investigated an SJA with the chevron orifice. The noise generated by an SJA is dependent on the shape and number of chevrons.

Zhang et al. [16] investigated numerically the impact of SJA parameters (orifice diameter and length and cavity diameter and height) on the characteristics of the synthetic jet. They demonstrated that the SPL increases with the orifice diameter. However, the dependence between SPL and orifice length was irregular, while Lesance et al. [10] and Kanase et al. [17] pointed out the proportional relationship between these values. The noise of the SJ was also investigated by others, such as Ikhlaq et al. [18], Jeyalingam and Jabbal [19], Paolillo et al. [20] and others.

This article focuses on determining the relationship between SJA parameters and SPL(A) and SJ velocity at different operating frequencies of the actuator. Changes to parameters such as the diameter and length of the orifice and the height of the cavity are taken into account. The research findings are presented in the context of other articles concerning the influence of actuator parameters on the noise generated by the SJA. The presented data expands knowledge in this area and can be used for designing SJAs. This investigation is broader than most of the existing studies because it discusses the influence of all significant geometrical features of the actuator on the generated noise, as indicated in the literature.

## 2. Materials and Methods

**Figure 1:**
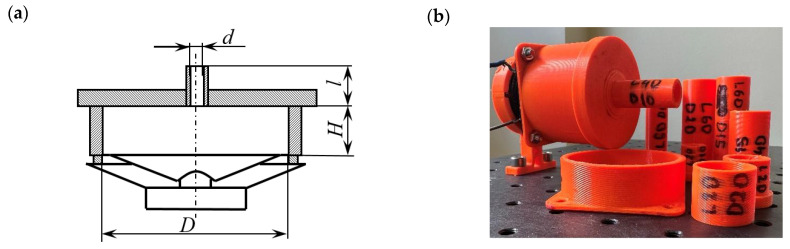
Schema of the synthetic jet actuator (**a**) and photo of the synthetic jet actuator’s elements (**b**).

The analyzed SJA consisted of a loudspeaker Monacor SP-6/4SQS with magnetic shielding (impedance Z=4 Ω, power rating P=3 W, frequency range f=3÷16,000 Hz) fixed to the actuator body with the internal diameter D=55 mm and cavity height H. The orifice of the actuator had an inner diameter d and a length l. The characteristic parameters of the actuator are presented in Figure 1a, and their values are in Table 1. Such choice of parameters allowed us to determine the impact of the orifice diameter (Case 1, Case 2, Case 3), orifice length (Case 4, Case 1, Case 5, Case 6) and cavity height (Case 7, Case 1, Case 8, Case 9) on the noise generated by the actuator. The cavity diameter was unchanged because it depends on the diameter of the loudspeaker used in the investigation.

The actuator was supplied with harmonic electrical current with a Rigol DG4162 (RIGOL Technologies, Inc., Beijing, China) wave-form generator and provided with a SeoUm Pa-940/2 amplifier (SeoUm, Seoul, Republic of Korea). The actuator input complex power was constant and equal to P=2 VA. The effective voltage, current, and frequency of the sinusoidal signal, which powered the SJA, were measured with a BENNING MM11 multimeter (Benning Power Electronics Sp. z o.o., Głosków, Poland). The actuator was tested in a frequency range f=50–500 Hz with frequency steps equal to 10 Hz. The actuator body (Figure 1b) was manufactured with 3D printing technology with polyethylene terephthalate glycol-modified (PET-G).

During the investigation, the SJ velocity and the SJA noise were measured. The velocity was measured with a MiniCTA 55T30 (Dantec Dynamics, Skovlunde, Denmark) hot-wire anemometer (HWA) with a single wire probe 55P16 (Dantec Dynamics, Skovlunde, Denmark), and an NI9215 (National Instruments, Austin, TX, USA) data acquisition device was used for the velocity measurements. The anemometer was calibrated within the range of 0.5–50 m∙s^−1^, with the use of a power-law relationship, and n=0.45 [21]. The measurement accuracy was ±6%. The velocity was measured in the centerline of the orifice at the orifice outlet area. For each actuator frequency f, the same number of points (n=10,000) was measured with the measurement’s frequency

(1) $$f_{m}=\frac{n}{1000}∙\frac{f}{100}$$

This means that, independently of the actuator frequency f, the velocity for 1000 periods was measured, and during each period, 100 measurement points were registered. The HWA probe was located in the axis of the orifice at a distance of 1 mm from the orifice.

The sound pressure level (SPL) of the SJ was measured with the Steinberg Systems SBS-SM-130C sound meter (IEC 61672-1 Class 2 standard [22]) with a measurement range of 30–130 dB(A), accuracy of ±1.4 dB and resolution of 0.1 dB. The sound meter was equipped with a ½’ microphone. Type A frequency weighting was applied to the SPL measurements—SPL(A). The SPL was measured according to ISO 3746:2010 [23] in a regular room. The sound meter was located 1 m from the actuator, and the background noise was at least 10 dB(A) lower than the noise generated by the actuator.

As the measurements were taken in a standard room, the meter was positioned in several different locations at the same height, 1 m from the actuator. The noise measurements at each location fell within the ±1.4 dB measurement error range. However, all measurements were taken from the same position of the sound meter. Noise measurements for the SJ in a regular room in accordance with ISO 3746:2010 [23] were also carried out in [13,14,15,24].

### Data Reduction

One of the most important parameters of SJs [25] is their characteristic velocity, which can be determined as:

(2) $$U_{0}=\frac{1}{T}\int_{0}^{T_{e}}u\left(t\right)dt$$

where T is a period of SJA operation $T=\frac{1}{f}$ [s], $T_{e}$ is a period of SJ expulsion, *u* is the instantaneous velocity at the orifice axis [m/s], and t is the time [s].

The impedance and power of the actuator were calculated as:

(3) $$Z=\frac{E}{I}$$

(4) P=E∙I

where E is the effective voltage [V], and I is the effective current (A). In this paper, the complex power was measured, and the phase shift between current and voltage was not included. However, for a characteristic frequency, the phase shift is equal to 0, and therefore the complex power and real power have the same value. The relationship between the phase shift and the actuator frequency was discussed more precisely by Gil and Smyk [26].

Additionally, the Reynolds number and the Stokes number were calculated as [27]:

(5) $$Re=\frac{U_{0}d}{\nu }$$

(6) $$St=\sqrt{\frac{2\pi fd^{2}}{\nu }}$$

where ν is the kinematic viscosity of air [m^2^/s]. All measurements were made at a humidity of 33–40%, at 21 °C.

## 3. Results and Discussion

### 3.1. The Flow Parameters

**Figure 2:**
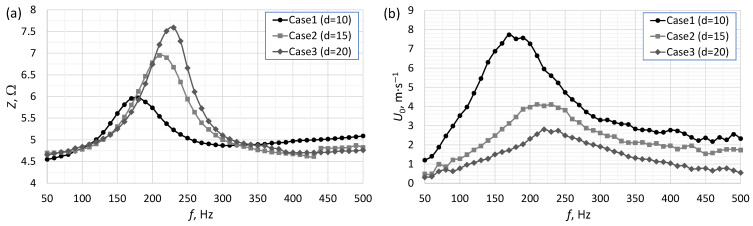
The impedance (**a**) and SJ velocity (**b**) as a function of the actuator frequency for an SJA with different orifice diameters.

**Figure 3:**
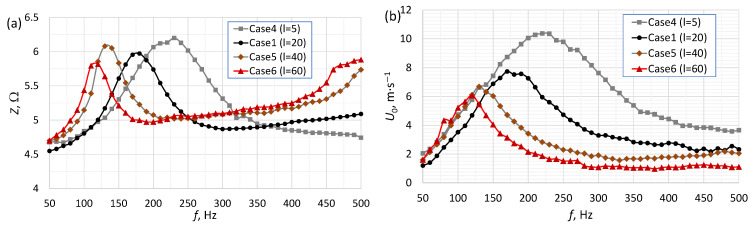
The impedance (**a**) and SJ velocity (**b**) as a function of the actuator’s frequency for an SJA with different orifice lengths.

**Figure 4:**
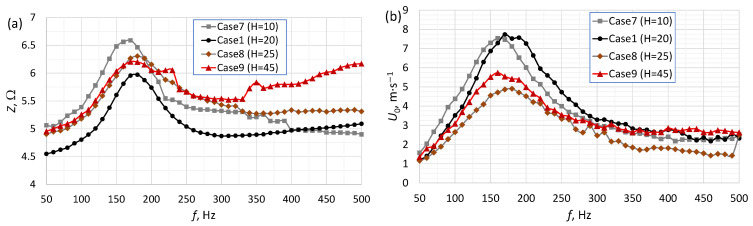
The impedance (**a**) and SJ velocity (**b**) as a function of the actuator’s frequency for an SJA with different cavity heights.

Figure 2, Figure 3 and Figure 4 present the impedance and the SJ velocity as a function of the actuator frequency for an SJA with different orifice diameters (Figure 2), orifice lengths (Figure 3) and cavity heights (Figure 4). The characteristic frequency of the actuator had a similar value (±10 Hz) regardless of whether it was determined based on impedance or SJ velocity. The value of the characteristic frequency for all cases is presented in Table 2. The characteristic frequency presented in Table 2 was designed based on SJ velocity. A change in any one of the parameters resulted in a change in the values of the characteristic frequency and SJ velocity.

The impedance value for the characteristic frequency was directly proportional to the orifice diameter (Figure 2a). The SJ velocity was higher the smaller the orifice, independently of the frequency of the actuator (Figure 2b). The decrease in the velocity value with the increase in the orifice value was an expected phenomenon. The flow forcing, i.e., the movement of the loudspeaker diaphragm, was constant regardless of the diameter of the orifice. Since the kinetic energy of the fluid (directly proportional to the flow field) flowing through the orifice should not change (neglecting losses), the velocity should decrease with an increase in the diameter. This dependence was shown by Gil and Strzelczyk [28]. However, they investigated actuators for the same effective voltage and a different power. However, Persoons et al. [29] showed that an increase in SJ velocity causes an increase in pressure losses in the orifice. Jacob et al. [30] also showed an increase in damping with a decrease in orifice diameter.

The impedance value for the characteristic frequency was similar (*Z* = 5.82–6.13 Ω), independently of the orifice length (Figure 3a). The SJ velocity for the characteristic frequency was higher for shorter orifices (Figure 3b). For high frequencies (*f* > 140 Hz), the velocity was always higher for shorter orifices, while for low frequencies (*f* < 140 Hz) the highest values were observed for different actuators depending on the frequency. Similar results were obtained by Gil and Strzelczyk [28]. They obtained the highest velocity for the longest orifice (*l* = 20 mm) for very low frequencies (*f* < 50 Hz). In this investigation, the velocity for the longest orifice (Case 6, l=60 mm) was the highest in the frequency range f=70–110 Hz. It is worth noting that the increase in the SJ velocity with the decrease in the orifice length for the characteristic frequency was suggested in [31,32] based on the heat transfer coefficient and Nusselt number value. It has been discussed in more detail in [33]. The investigation presented by Jain et al. [34] suggests that there is an optimum orifice height at which the maximum velocity can be achieved (see [35]). The relationship between the aspect ratio l/d and the characteristic diameter is presented later in this paper.

The impedance value for the characteristic frequency was inversely proportional to the cavity height—except for Case 1 (Figure 4a), for which the lowest impedance value was measured (Z=5.95 Ω). The SJ velocity was the highest for Case 1 at frequency f>160 Hz and for Case 7 at frequency f≤160 Hz. The velocity for the characteristic frequency was similar in for 1 and Case 7 ($∆U_{0\_17}=0.21$ m/s). The lowest velocity throughout the entire frequency range was obtained for Case 9 (H=45 mm). Previous works indicated the relationship between the height of the actuator chamber and the characteristic frequencies [28,36]. Despite this, no models were found that would allow the natural frequency to be calculated while taking this parameter into account. This problem was discussed in more detail in [36].

**Figure 5:**
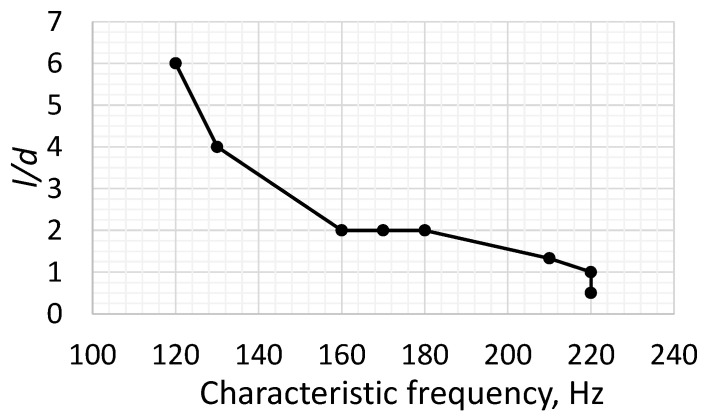
Dependence of characteristic frequency on *l*/*d*.

Jani et al. [34] showed that the cavity shape has an impact on the shape of the velocity profile and therefore on the characteristic velocity. On the other hand, Jani et al. [34] and Kordík and Trávníek [37] indicated a lack of correlation between the chamber height and the first characteristic frequency. The data presented in Table 2 indicates the existence of such a relationship, albeit a small one. A similar conclusion was also drawn by Chaudhari et al. [38]. The increase in SPL(A) with the cavity size may be caused by the extension of the sound propagation time and the appearance of flow turbulence in the cavity. However, it is common to see a relationship between the orifice size and the characteristic frequency, which is also visible in Figure 5. For Case 3 (l/d=1) and Case 4 (l/d=4), the determined characteristic frequency was equal to 220 Hz but was probably caused by a large step of the frequency value during the measurements. Generally, the larger the *l*/*d* value, the higher the first characteristic frequency (Figure 5). Analytical methods for determining the characteristic frequency of an actuator suggest a similar relationship between these values [37,39,40].

### 3.2. Noise Measurements

**Figure 6:**
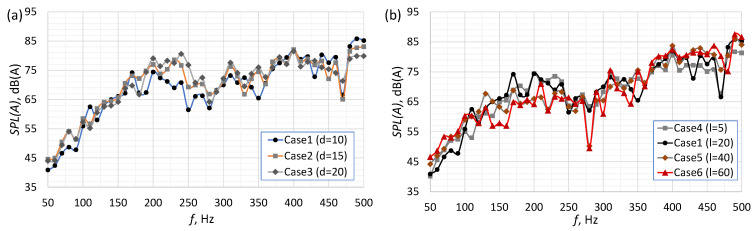

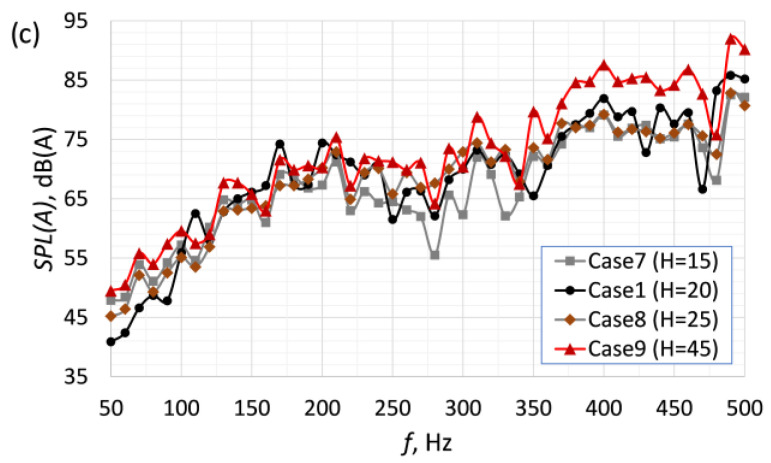
The SPL(A) for SJAs with different (**a**) orifice diameters, (**b**) orifice lengths, (**c**) cavity heights.

Figure 6 shows the SPL(A) for SJAs with different orifice diameters, orifice lengths, and cavity heights. The SPL(A) increased with the frequency in all investigated cases. However, a small local decrease in SPL(A) can be observed near 250 Hz. The shape of the SPL(A) as a function of the frequency is similar to the A-weighting curve. However, the A-weighting adjustment factor at 50 Hz was equal to −30.2 Hz and at 500 Hz was equal to −3.2 Hz. The differences between the SPL(A) values at 50 Hz and 500 Hz were higher than 30 dB; so, they were not just the result of applying the A-weighting curve. It should be noted that the obtained curves differed significantly in shape and course from the data presented by Zhang et al. [16].

**Figure 7:**
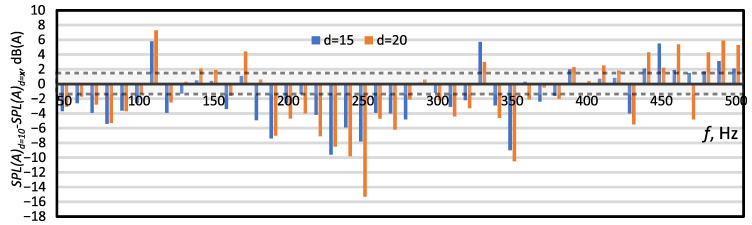
The difference between the SPL(A) for Case 1 (*d* = 10) and the SPL(A) for Case 2 (*d* = 15, blue poles) and Case 3 (*d* = 20, orange poles). The dashed lines and the shaded area between them indicate the area of measurement uncertainty, ±1.4 dB.

**Figure 8:**
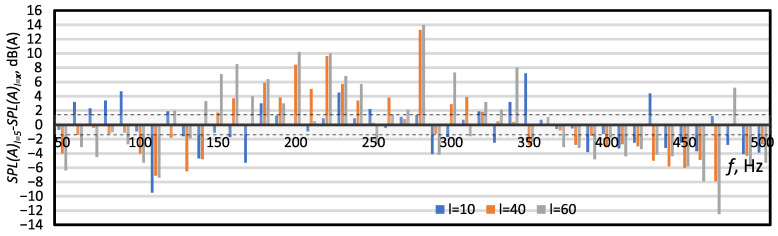
The difference between the SPL(A) for Case 4 (*l* = 5) and the SPL(A) for Case 1 (*l* = 10, blue poles), Case 5 (*l* = 40, orange poles) and Case 6 (*l* = 60, grey poles). The dashed lines and the shaded area between them indicate the area of measurement uncertainty, ±1.4 dB.

**Figure 9:**
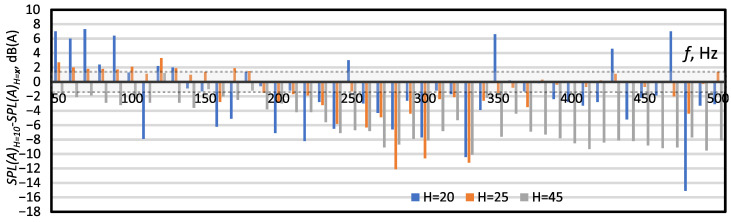
The difference between the SPL(A) for Case 7 (*H* = 10) and the SPL(A) for Case 1(*H* = 10, blue poles), Case 8 (*H* = 25, orange poles) and Case 9 (*H* = 45, grey poles). The dashed lines and the shaded area between them indicate the area of measurement uncertainty ±1.4 dB.

The curves presented in Figure 6 are difficult to interpret due to a large number of changes in the relative sound levels. First, we can observe an increase in the generated noise with an increasing frequency, for f<200 Hz, then a slight decrease in the noise level for frequencies in the 200<f<350 Hz range and a further increase in the SPL(A) value for f>350 Hz. For all cases studied, a local decrease was also observed for the frequency of 470 or 480 Hz. To better compare the changes between individual cases, the graphs presented in Figure 7, Figure 8 and Figure 9 were prepared. Figure 7Figure 8 and Figure 9 show the difference in SPL(A) between the actuator with the smallest orifice diameter, orifice length and cavity height, and other cases. The graphs were drawn in such a way as to show how increasing the value of individual parameters affected the SPL(A) value. Additionally, a grey area was added to all graphs to indicate measurement uncertainty.

Figure 7 presents differences in SPL(A) levels between Case 1 and cases with larger orifice diameters (Case 2 and Case 3). The increase in the orifice diameter caused an increase in the SPL(A) level for most frequencies less than 390 Hz. However, for f≥440 Hz the relationship was reversed. Increasing the orifice diameter caused a decrease in the SPL(A). The dependence of the SPL(A) and the diameter on the actuator orifice was investigated in [10,17,41]. These papers consistently indicated that the larger the orifice diameter, the higher the SPL(A) level generated by the SJA. On the other hand, Zhang et al. [16] simulated the SJ and showed that the SPL increased with the orifice diameter but only for the characteristic frequency. Table 2 shows that this was also true in this investigation. The SPL(A) for Case 2 in resonance frequency was about 0.4 dB lower than for Case 1 in resonance frequency. However, the accuracy of the measurement was ±1.4 dB. The values should therefore be assumed to be similar. The area of measurement accuracy is indicated by the dashed lines and the shaded area between them.

Figure 8 presents differences in SPL(A) levels between Case 4 and cases with a longer orifice (Case 1, Case 5 and Case 6). The dependence of the SPL(A) level on the orifice length is not clear. However, two characteristic areas can be distinguished in Figure 8: first, for a frequency in the range of 180≤f≤240 Hz (characteristic frequency areas), where the longer the orifice, the lower the SPL(A); and second, for a frequency in the range of 370≤f≤500 Hz, where generally, the longer the orifice, the higher the SPL(A). Kanase et al. [17] and Lesance et al. [10] showed that the longer the orifice, and the lower the generated noise. Bhapkar et al. [41] reached different conclusions. Based on studies [10,17], it should be noted that Bhapkar et al. [41] made an error of inference during their research. By maintaining the same shape factor, they estimated that the orifice length influenced the generated noise, not the orifice diameter, which in this case turned out to be the decisive factor. The results presented in the above papers coincide with the results of the presented research in the range close to the characteristic frequency, 180≤f≤240. On the other hand, Zhang et al. [16] showed that the SPL can change irregularly with the increasing in the orifice length for the characteristic frequency. However, the results presented by Zhang et al. [16] are numerical, and the results presented by Kanase et al. [17] and Lesance et al. [10] are experimental.

Figure 9 presents differences in the SPL(A) levels between Case 7 and cases with a higher cavity (Case 1, Case 8, and Case 9). The SPL(A) was generally the lower, the higher the cavity, for f≥190 Hz. There were deviations from this rule, for example for the frequency of 500 Hz, at which the lowest sound level was obtained for H=45 mm and then for H=20 mm and H=10 mm, and the loudest noise was recorded for H=25  mm. However, for most measurement points, the lowest SPL(A) level was obtained for the highest cavity. For the frequency f<190 Hz, the relationship between cavity height and SPL(A) level was not clear. The impact of the cavity size and generated noise is very often overlooked. The SPL has been more often tested for generators with a different design [42,43] or a different orifice shape [17,24]. Zhang et al. [16] showed that the SPL decreased with the increase in cavity height. This is inconsistent with the observation that we made. However, considering the entire measurement range, it was not a general trend nor a constant relationship. Zhang et al. [16] investigated SJAs numerically, and we presented in our article measured data. However, it should be noted that the measurements were carried out rather than in an anechoic chamber, in a laboratory room. Due to this, they may have been affected by external disturbances that the authors of the paper did not notice or predict, despite taking special care during the measurements.

### 3.3. Nois vs. Velocity

**Figure 10:**
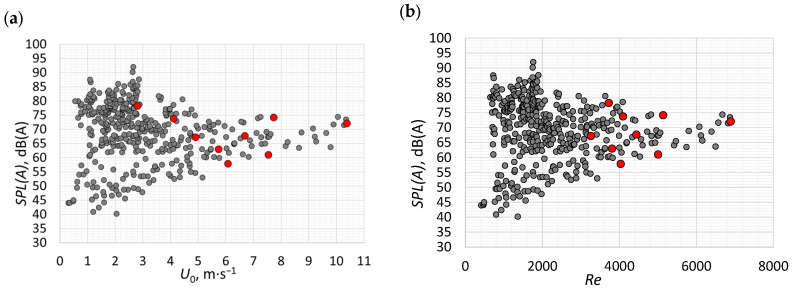
The SPL(A) vs. SJ velocity (**a**) and Reynolds number (**b**)—red dots indicate the values obtained for the characteristic frequencies of the actuators (Table 2).

Many authors reported a relationship between SJ velocity and the noise generated by actuators. This relationship was presented by Gil et al. [11] and Smyk et al. [14] for different actuators. However, the investigated actuators operated at the characteristic frequencies, and the increase in the velocity was obtained by increasing the supply power. Figure 10 presents the SPL(A) generated by the investigated actuators as a function of SJ velocity and Reynolds number. The noise generated at the characteristic frequency is marked by red dots. As expected, given that the Reynolds number is a function of velocity, the two relationships are similar. However, the noise level is usually related to the flow velocity rather than to the Reynolds number. Therefore, the results are here discussed based on Figure 10a. The corresponding Reynolds number values are presented in brackets throughout.

It should be noted that most of the measurement points are concentrated in the area including velocities of less than 4 m/s and noise greater than 65 dB(A). This cannot be seen as a trend, and our task was only to obtain a random distribution of measurements resulting from the adopted dimensions and parameters of the actuator. It should be noted that for the highest velocities ($U_{0}>9$ m/s and Re>6000), the obtained SPL(A) was not the highest. In this region, the SPL(A) was in the range of 62–75 dB(A), while the highest noise obtained was over 90 dB(A). In the authors’ opinion, this means that the dependence of the noise generated by the actuator on the SJ velocity is not a simple relationship, and an increase in speed does not necessarily cause an increase in the noise level—this does not apply when comparing the SPL(A) levels obtained from one generator operating at a constant frequency but with different supply power [11,14]. However, Gil et al. [11] showed that even when comparing the noise of actuators operating at the characteristic frequency for the same power, the noise–velocity relationship is not a simple linear relationship. They pointed out that the d/D ratio is very important in assessing the generated noise. Nevertheless, the scatter of the results was quite large. The number of actuator variants tested in this article made it impossible to reliably determine the relationship between noise and the d/D ratio.

### 3.4. Actuator Manufacturing Technology

This article used actuators manufactured with 3D printing technology. This technology is becoming increasingly widely used in industry and science but is still not the default solution in SJ research. For this reason, the cited articles were reviewed, and Table 3 lists the materials used to make the actuator body in the individual investigations. A significant part of the cited papers did not provide information on the material from which the actuator was made, and in one work the material classification was made on the basis of a photo of the test stand (Jeyalingam and Jabbal [19]).

The materials most often used in the construction of actuators are aluminum alloys and poly(methyl methacrylate) (PMMA). These are widely available materials, easy to process and with a uniform structure. They do not allow air or water to pass through. Three-dimensional prints made using fused deposition modeling (FDM) technology are fluid-permeable and can allow air to pass through in the event of large pressure differences. However, the authors found no articles examining this phenomenon. These prints are also characterized by higher surface roughness than machined surfaces. It should be noted that an increased surface roughness in the SJA orifice may affect the jet discharge characteristics. However, the characteristics shown in Figure 2, Figure 3, Figure 4 and Figure 5 overlap in their trends relative to other data [28,36].

Roughness can also affect the noise level. However, for all variants tested in this paper, the print parameters were the same. It should therefore be assumed that if the technology had an impact on the measured parameters, then it had the same impact in all cases.

It should also be noted that although the prints were not printed as filled, the walls consisted of two full outlines. Considering the low pressures in the actuator and the speed of the process, it should be assumed that there was no flow through the actuator walls.

## 4. Conclusions

In this paper, the impact of the orifice diameter, orifice length, and cavity height on SJ velocity and SPL(A) was analyzed. Based on the analysis of the measurement results and a literature review, it can be stated that the following connections occur for acoustic SJAs operated in (or near) the characteristic frequency:

- It was shown that generally, the higher the actuator chamber, the higher the noise, although this was not a constant trend across the entire frequency range tested. This observation is inconsistent with the literature data on orifice diameter.
- The SJ velocity increases with the decreasing orifice length, which is caused by the flow losses increasing with the orifice length.
- No relationship was observed between cavity height and SJ velocity. However, different velocities were observed for different cavity heights (Figure 4). It should therefore be emphasized that such a relationship probably exists, but it was not possible to establish it in this paper. Mathematical models of the resonance frequency do not describe this relationship, even though it has already been demonstrated in the literature.
- An inversely proportional relationship exists between the orifice size (*l*/*d*) and the characteristic frequency (Figure 5).
- The SPL(A) increases with the increasing orifice diameter; this relationship is not entirely clear, as increasing the orifice causes a lower average velocity, which should increase noise. However, increasing the orifice diameter may cause an increase in volumetric flow, which may cause an increase in the vorticity behind the orifice and, consequently, in noise; further investigation of this aspect is recommended.
- The SPL(A) decreases with the increasing orifice length, which is related to the drop in velocity in an orifice.
- The SPL(A) increases with the increasing cavity height; this effect may be caused by improved resonance conditions in the chamber (increasing the chamber height causes a decrease in the Helmholtz resonance frequency) and may promote the formation of turbulence in the chamber.

Additionally, the relationships between the individual parameters across the full range of tested frequencies were discussed. The presented data could help to minimize noise generated by SJAs and could be useful for conducting further research within the presented range. Based on the above conclusions and the literature review, it should be noted that this subject has not yet been fully explored. Further solutions should be sought to reduce the noise generated by SJAs, while maintaining the flow parameters. There are also no mathematical models that accurately describe the relationship between noise and actuator parameters. It is therefore reasonable to use numerical simulations to investigate the relationships described in the article more precisely.

## Figures and Tables

**Table 1:** The parameters of the SJA.

	H [mm]	l [mm]	d [mm]	l/d	d/D	H/d
Case 1	20	20	10	2	0.18	2
Case 2	20	20	15	1.33	0.27	1.33
Case 3	20	20	20	1	0.36	1
Case 4	20	5	10	0.5	0.18	2
Case 5	20	40	10	4	0.18	2
Case 6	20	60	10	6	0.18	2
Case 7	10	20	10	2	0.18	1
Case 8	25	20	10	2	0.18	2.5
Case 9	45	20	10	2	0.18	4.5

**Table 2:** Parameters of the SJ for the characteristic frequency.

	f [Hz]	SPL (A) [dB(A)]	*Re*	*St*
Case 1	170	74.2	5135	84.2
Case 2	210	73.8	4093	140.4
Case 3	220	78.3	3725	191.6
Case 4	220	72.1	6890	95.8
Case 5	130	67.7	4441	73.6
Case 6	120	57.9	4034	70.8
Case 7	160	61	5006	81.7
Case 8	180	67.2	3258	86.7
Case 9	160	63	3809	81.7

**Table 3:** Materials used to manufacture actuator bodies.

Body Material	Articles
Aluminum alloy	Gil et al. [5]; Gil et al. [11]; Gil and Wilk [13]; Paolillo et al. [20]
PMMA	Smyk et al. [14]; Jeyalingam and Jabbal [19] *; Bhapkar et al. [24]; Singh et al. [31]; Singh et al. [32]; Chaudhari et al. [38]
Cavity from PMMA and orifice manufactured with 3D printing technology	Smyk and Markowicz [15]
Some metal	Ikhlaq et al. [18]
Manufactured with 3D printing technology	Gil and Smyk [26]; Smyk et al. [36]

* Material determined based on the photo included in the article.

## Data Availability

The data that support the findings of this study is available from the corresponding author upon reasonable request.
